# On Acute Rheumatism

**Published:** 1807-11

**Authors:** 


### To the Editors of the Medical and Physical Journal.

GENTLEMEN,

THE 80th No. of your Journal conveyed some ingenious remarks by Mr. Harrold, on a Case designated "Acute Rheumatism terminating in suppuration," which you were so obliging as to insert for me in a preceding number. Begging Mr. Harrold's pardon for my long delay, I embrace this the most convenient opportunity that has since offered, to notice those remarks. This was a case of acute migrating inflammation, which, after the patient had complained several days of pain and stiffness, principally in the wrists, was ushered in by high inflammatory excitement of the system. It appeared on one knee, then attacked successively the other knee, the ankles, the arms, wrists, and fingers, the clavicle and shoulder, and finally the intercostal muscles, diaphragm, and the bowels; terminating fatally on the 12th day after its invasion. The subject of the disease was a thin, spare lad, of inflammatory habit, who became ill after having lain on the ground, while heated by exercise, in the month of October. The general expression of the disease so strongly represented the rheumatic type to my view, that there appeared to me nothing ambiguous in it, excepting a greater disposition to run into suppuration than what genuine rheumatic inflammation usually has. My own experience (not the most extensive to be sure) undoubtedly had not furnished me with a parallel exception; but, considering the remote cause of the disease, the symptoms in their origin and progress, the series of parts attacked, the tendency to metastasis, and the mode of fatal termination by attacking the respiratory muscles and the intestines; I did not conceive that this tendency to suppuration, partial as it was, affected essentially the character of the disease. In its general contour it must be allowed to represent, with tolerable precision, the prototype given by Cullen in these words: Rheumatismus.——Morbus ab externa, et plerumque evidente causa; pyrexia; dolor circa articulos, musculorum tractum sequens, genua et reliquos majores, potius quam pedum vel manuum articulos infestans, calore externo auctus. The specific inflammations do not always keep within their proper limit, as appears occasionally to be the case in scrophulous inflammation; in erysipelas, the inflammation of small-pox, chicken-pox, &c. The case in question I had considered as an example of such deviation, not admitting of doubt, till it was suggested by Mr. Harrold. That Gentleman endeavours in the paper above referred to, to shew that the specific character of the inflammation here, was rather that of the true uncombined phlegmon than of legitimate rheumatism. The cases which fell under his observation, to which he alludes, which he collates with mine, and upon which he seems to ground his arguments, arguments, had, very probably, no affinity to rheumatism; but that this was also the state of the case I have described, requires some further confirmation than what Mr. H. has advanced. The case was a notable one, and of importance in a pathological view: it ranks with those which Darwin, Kirkland, and others, have deemed to be instances of rheumatism inducing suppuration. And it was brought forwards by me as a well marked instance of such an unusual termination or combination: an outline only was therefore obviously intended to be sketched sufficiently to identify the primary disease. While I contend that the essence of the case was genuine rheumatism, I am principally solicitous to ascertain, whether it is to be considered an admitted fact, in the history of that disease, that one of its terminations is in suppuration?—A question I should be highly gratified to see decided upon more ample evidence than is afforded by our systematic authors.

